# Comparison of Diagnostic Algorithms for Detecting Toxigenic *Clostridium difficile* in Routine Practice at a Tertiary Referral Hospital in Korea

**DOI:** 10.1371/journal.pone.0161139

**Published:** 2016-08-17

**Authors:** Hee-Won Moon, Hyeong Nyeon Kim, Mina Hur, Hee Sook Shim, Heejung Kim, Yeo-Min Yun

**Affiliations:** 1 Department of Laboratory Medicine, Konkuk University School of Medicine, Seoul, Korea; 2 Department of Laboratory Medicine, Yonsei University College of Medicine, Seoul, Korea; Cornell University, UNITED STATES

## Abstract

Since every single test has some limitations for detecting toxigenic *Clostridium difficile*, multistep algorithms are recommended. This study aimed to compare the current, representative diagnostic algorithms for detecting toxigenic *C*. *difficile*, using VIDAS *C*. *difficile* toxin A&B (toxin ELFA), VIDAS *C*. *difficile* GDH (GDH ELFA, bioMérieux, Marcy-l’Etoile, France), and Xpert *C*. *difficile* (Cepheid, Sunnyvale, California, USA). In 271 consecutive stool samples, toxigenic culture, toxin ELFA, GDH ELFA, and Xpert *C*. *difficile* were performed. We simulated two algorithms: screening by GDH ELFA and confirmation by Xpert *C*. *difficile* (GDH + Xpert) and combined algorithm of GDH ELFA, toxin ELFA, and Xpert *C*. *difficile* (GDH + Toxin + Xpert). The performance of each assay and algorithm was assessed. The agreement of Xpert *C*. *difficile* and two algorithms (GDH + Xpert and GDH+ Toxin + Xpert) with toxigenic culture were strong (Kappa, 0.848, 0.857, and 0.868, respectively). The sensitivity, specificity, positive predictive value (PPV) and negative predictive value (NPV) of algorithms (GDH + Xpert and GDH + Toxin + Xpert) were 96.7%, 95.8%, 85.0%, 98.1%, and 94.5%, 95.8%, 82.3%, 98.5%, respectively. There were no significant differences between Xpert *C*. *difficile* and two algorithms in sensitivity, specificity, PPV and NPV. The performances of both algorithms for detecting toxigenic *C*. *difficile* were comparable to that of Xpert *C*. *difficile*. Either algorithm would be useful in clinical laboratories and can be optimized in the diagnostic workflow of *C*. *difficile* depending on costs, test volume, and clinical needs.

## Introduction

*Clostridium difficile* is a primary pathogen causing antibiotic-associated colitis and responsible for 15–25% of cases of nosocomial antibiotic-associated diarrhea [1, 2]. The clinical manifestations of *C*. *difficile* infection (CDI) range from mild diarrhea to pseudomembranous colitis and cannot be distinguished with those from other causes, making laboratory confirmation essential [1, 3].

The two reference methods for detecting toxigenic *C*. *difficile* are the toxigenic culture and cell cytotoxicity assay [4, 5]. However, these assays have limitations such as long turnaround time, technical complexity, and lack of standardization [6]. In practice, the most widely used technique for detecting toxigenic *C*. *difficile* had been toxin detection using enzyme immunoassay (EIA). It is fast and inexpensive; however, its low sensitivity is a limitation for its use [7, 8].

Glutamate dehydrogenase (GDH) is a common antigen of the *C*. *difficile* cell wall and GDH testing detects both non-toxigenic and toxigenic *C*. *difficile*. It shows high sensitivity and negative predictive value (NPV), but it should be paired with a test detecting toxin [1, 9]. Recently, nucleic acid amplification testing (NAAT), including rapid, ready-to-use molecular assays, has been introduced and shown high sensitivity and specificity [9–11]. However, its cost-effectiveness as well as its usefulness as a stand-alone test is still questionable [1, 4, 9–13]. Since every single test has some limitations, many experts and guidelines recommend the approaches using multiple tests (multistep algorithms) for the detection of toxigenic *C*. *difficile* [1, 9, 11, 13–15].

This study aimed to compare the current, representative diagnostic algorithms for detecting toxigenic *C*. *difficile*, using VIDAS *C*. *difficile* toxin A&B, VIDAS *C*. *difficile* GDH (bioMérieux, Marcy-l’Etoile, France), and Xpert *C*. *difficile* (Cepheid, Sunnyvale, California, USA).

## Materials and Methods

### Clinical samples

This *in vitro* study was approved by the Institutional Review Board of the Konkuk University Medical Center, Seoul, Korea (a tertiary referral hospital with 900 beds). From January 2015 to April 2015, we collected 271 consecutive, remnant diarrheal stool samples submitted to the clinical microbiology laboratory from patients admitted to our hospital.They included 258 adults and 13 children (male 148, female 123). Since we used remnant samples after routine tests and the data were analyzed anonymously, informed consent was exempted. As a routine practice of *C*. *difficile* testing in our hospital, we performed toxigenic culture and VIDAS *C*. *difficile* toxin A&B (bioMérieux) simultaneously. The VIDAS *C*. *difficile* GDH (bioMérieux) and Xpert *C*. *difficile* (Cepheid) were additionally performed in the same samples. Duplicated samples from the same patients and from patients on treatment for CDI were excluded. The samples were tested within 2 hours of collection; otherwise, they were kept at 2–8°C for up to 2 days.

### Toxigenic culture of *C*. *difficile*

The alcohol-shocked stool samples were inoculated onto on a chromogenic agar plate (chromID CD agar, bioMérieux), and the plates were incubated at 37°C under anaerobic conditions (Forma Anaerobic System; Thermo Fisher Scientific, Waltham, MA, USA) for 48 hrs. The isolates of typical morphology with gray-to-black colonies with irregular or smooth borders suspicious for *C*. *difficile* were initially investigated by Gram staining and finally identified by the matrix-assisted laser desorption/ionization time-of-flight (MALDI-TOF) using VITEK MS system (bioMérieux). Isolates of *C*. *difficile* were examined for toxin production using in-house PCR as described previously [16, 17].

### VIDAS *C*. *difficile* toxin A&B and VIDAS *C*. *difficile* GDH

Stool samples were tested for toxin and GDH using VIDAS *C*. *difficile* toxin A&B and VIDAS *C*. *difficile* GDH kit (bioMérieux), respectively, according to the manufacturer's instructions. The assay principle combines a two-step enzyme immunoassay sandwich method with a final fluorescent detection (enzyme-linked fluorescence immunoassay, ELFA). An aliquot of liquid stool was added in the specific diluent, and supernatant after centrifugation was tested. The VIDAS *C*. *difficile* toxin A&B is completed within 75 minutes, and results are reported as negative, equivocal, or positive with cut-offs of 0.13 and 0.37 test value (TV). There were 10 equivocal results in our study and they were considered negative for the performance calculations in this study. The VIDAS *C*. *difficile* GDH is completed within 50 minutes, and results are reported as negative or positive with a cut-off of 0.10 of TV.

### Xpert *C*. *difficile*

Xpert *C*. *difficile* system (Cepheid) is an automated, real-time multiplex PCR assay using disposable cartridge. This assay detects toxin B (*tcdB*), binary toxin (*cdt*), and a point mutation associated with PCR ribotype 027. The result of the toxin B target means the presence of toxigenic *C*. *difficile*. The results of other targets indicate additional information regarding ribotype 027. The invalid result is reported when sample processing control is failed, indicating that the sample is not properly processed or PCR is inhibited. Using a maximum valid cycle threshold setting of 37 for *tcdB*, the limit of detection point estimate for toxigenic *C*. *difficile* is 1,657 CFU/swab (95% confidence interval [CI], 1,157–3,561 CFU/swab), according to the manufacturer’s insert.

### Diagnostic algorithms

**Fig 1:**
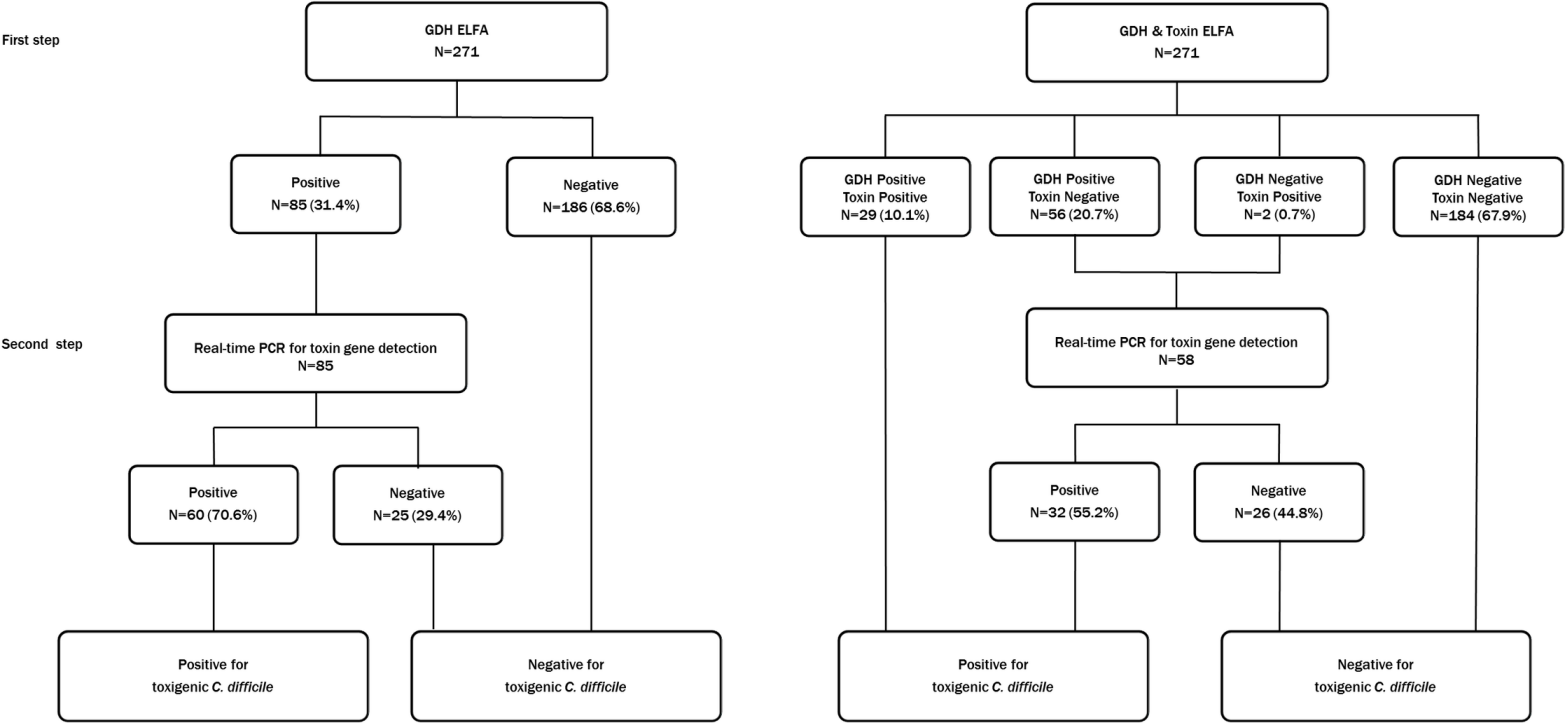
The diagnostic algorithms and distribution of samples in each step of algorithms. GDH-based 2-step algorithm, in which GDH ELFA is initially performed and followed by toxin gene detection using the Xpert *C*. *difficile* (GDH + Xpert) (left) and combined algorithm of toxin ELFA, GDH ELFA and the Xpert *C*. *difficile*, in which initial screening is performed with GDH ELFA and toxin ELFA simultaneously and followed by the Xpert *C*. *difficile* in discordant samples (GDH + Toxin + Xpert) (right). One invalid result by the Xpert *C*. *difficile* is included.

We selected the following diagnostic algorithms using VIDAS *C*. *difficile* toxin A&B (toxin ELFA), VIDAS *C*. *difficile* GDH (GDH ELFA), and Xpert *C*. *difficile* (**Fig 1**); GDH-based 2-step algorithm, in which GDH ELFA is initially performed and followed by toxin gene detection using Xpert *C*. *difficile* (GDH + Xpert)[11] and combined algorithm of GDH ELFA, toxin ELFA, and Xpert *C*. *difficile*, in which the initial screening is performed with GDH ELFA and toxin ELFA simultaneously and followed by Xpert *C*. *difficile* in discordant samples (GDH + Toxin + Xpert)[9]. The average costs and assay times were simulated based on generally used reagent costs [18, 19] and assay times shown in insert papers of individual assays.

### Statistical analysis

The sensitivity, specificity, positive predictive values (PPV), and NPV with 95% CI of each assay and algorithm were determined based on the results of toxigenic culture as a gold standard. McNemar’s test was used for the statistical differences of sensitivity and specificity between assays and algorithms and chi-square test for the statistical differences of PPV and NPV between them. Agreement between assays were determined using Cohen’s Kappa (agreement: < 0.20, none; 0.21–0.39, minimal; 0.40–0.59, weak; 0.60–0.79, moderate; 0.80–0.90, strong; > 0.90, almost perfect) [20]. Statistical analysis was performed using MedCalc Statistical Software (version 12.3.0, MedCalc Software, Mariakerke, Belgium) and IBM SPSS Statistics 22.0 (IBM Corporation, Armonk, NY, USA).

## Results

The result of each assay and algorithm was compared with that of toxigenic culture (**Table 1)**. The agreement of toxin ELFA and GDH ELFA with toxigenic culture were weak and moderate (Kappa, 0.564 and 0.678, respectively). The agreement of the Xpert *C*. *difficile* and the two algorithms (GDH + Xpert and GDH + Toxin + Xpert) with toxigenic culture were strong (Kappa, 0.848, 0.857, and 0.868, respectively).

**Table 1:** Comparison of each assay and algorithm with toxigenic culture.

Toxigenic culture	Toxin ELFA		GDH ELFA		Xpert *C*. *difficile*		GDH + Xpert		GDH + Toxin + Xpert	
	Positive	Negative	Positive	Negative	Positive	Negative	Positive	Negative	Positive	Negative
Positive	27	28	53	2	52	3	51	4	52	3
Negative	4	212	32	184	11	204	9	206	9	206
Total	31	240	85	186	63	207	60	210	61	209
Kappa	0.564		0.678		0.848		0.856		0.868	
95% CI	0.433–0.695		0.581–0.775		0.772–0.925		0.781–0.932		0.796–0.941	

Based on toxigenic culture, toxin ELFA showed significantly lower sensitivity and NPV than the other assays and algorithms (*P* < 0.0001, both), while GDH ELFA showed significantly lower specificity (*P* < 0.0001) and PPV (*P* = 0.0113, 0.0079, 0.0030 and 0.0094, respectively) than toxin GDH, Xpert *C*. *difficile*, GDH + Xpert, and GDH + Toxin + Xpert (**Table 2**). There were no significant differences between the Xpert *C*. *difficile*, GDH + Xpert, and GDH + Toxin + Xpert in sensitivity, specificity, PPV, and NPV. Their sensitivity and specificity ranged from 95% to 97%. Their PPV ranged from 82% to 85%, and their NPV were all above 98%.

**Table 2:** Overall performance of each assay and algorithm based on toxigenic culture.

	Toxin ELFA	GDH ELFA	Xpert *C*. *difficile*	GDH+ Xpert	GDH+Toxin+ Xpert
Sensitivity	**49.1%**	96.4%	94.6%	96.7%	94.5%
95% CI	(35.4% - 62.9%)	(87.5% - 99.6%)	(84.9% - 98.8%)	(82.4% - 97.9%)	(84.8% - 98.9%)
Specificity	98.2%	**85.2%**	94.9	95.8%	95.8%
95% CI	(95.3% - 99.5%)	(79.7% - 89.6%)	(91.0% - 97.4%)	(92.2% - 98.1%)	(92.2% - 98.1%)
PPV	87.1%	**62.4%**	82.5%	85.0%	82.3%
95% CI	(70.2% - 96.4%)	(51.2% - 72.6%)	(70.9% - 90.9%)	(73.4% - 92.9%)	(73.8% - 93.2%)
NPV	**88.3%**	98.2%	98.6%	98.1%	98.5%
95% CI	(83.6% - 92.4%)	(96.2% - 99.9%)	(95.8% - 99.7%)	(95.2% - 99.5%)	(95.9% - 99.7%)

**Fig 1**shows the distribution of samples in each step of algorithms. The proportion of samples reported at the first step of algorithm was 68.6% (186/271) by GDH + Xpert and 78.6% (213/271) by GDH + Toxin + Xpert (**Fig 1 and Table 3**). Based on our distribution of samples, estimated average costs and assay times are also shown in **Table 3**. Cost per sample included reagent costs only and other costs like labor or quality control were not included. The assay time is calculated when GDH and toxin assays are performed concurrently in separate racks. The assay time per 30 samples was simulated based on capacity of our laboratory which has 1 GeneXpert with 4 cartilages spaces and 1 VIDAS with 5 racks (30 samples load).

**Table 3:** The reagent costs and assay times of the two diagnostic algorithms of *C*. *difficile* compared with those of Xpert *C*. *difficile*.

	Samples reported, n. (%)	Cost per sample, USD	Assay time per sample	Assay time per 30 samples		
				Samples	N. of runs	Time
GDH + Xpert						
*1 step*	186 (68.6%)	10	50 min	30	1	50 min
*2 step*	85 (31.4%)	10 + 50	50 + 45 min	9	3	135 min
Average		25.7	64.1 min	Total		185 min
GDH + Toxin + Xpert						
*1 step*	213 (78.6%)	20	75 min	30	2	100 min
*2 step*	58 (21.4%)	20 + 50	75 + 45 min	6	2	90 min
Average		30.7	84.6 min	Total		190 min
Xpert *C*. *difficile* only						
*1 step*	271 (100.0%)	50	45 min	30	8	360 min

## Discussion

The best laboratory method for diagnosis of CDI remains controversial. The widely used assays have been *C difficile* toxin EIA, GDH EIA, and NAAT, and they have different targets. The toxin EIA directly detects free toxin in feces, GDH test detects a common antigen produced by *C difficile*, and NAAT detects toxin genes but not free toxin [4, 21]. Although the commercially available toxin and GDH EIA are simple, rapid, and inexpensive, they are not suitable as stand-alone tests due to suboptimal sensitivity and specificity, respectively [1, 15]. Accordingly, several two- or three-step algorithms have been proposed, and more various algorithms are now in use in routine laboratories [1, 4, 9, 11, 13–15]. We wanted to compare the representative diagnostic algorithms for detecting toxigenic *C*. *difficile* using updated, automated, and widely used assays in clinical samples. Compared with toxigenic culture, two diagnostic algorithms, GDH + Xpert and GDH + Toxin + Xpert, showed strong agreements that are comparable to that of the Xpert *C*. *difficile* (**Table 1**).

In this study, toxin ELFA showed significantly lower sensitivity (49.1%) and NPV (88.3%) with comparable specificity and PPV to the other assays and algorithms. This finding is generally in line with most previous reports. Toxin EIA including ELFA, however, have shown widely varying sensitivity (32–99%) and specificity (65–100%), depending types of assays and used reference methods [1, 9]. Although toxin EIA or toxin PCR after GDH assay had been recommended in early guidelines [14], the algorithm screening by GDH ELFA confirmed by toxin ELFA also showed low sensitivity (sensitivity 47.3%, CI, 33.9–61.2 and specificity 98.6%, CI, 95.7–99.6, data not shown) similar to that of toxin ELFA only. There is equivocal zone in toxin ELFA and there were 10 equivocal results in our study. Among them, 4 samples were toxigenic culture positive and 5 samples were Xpert *C*. *difficile* positive. When we considered equivocal results as positive category, toxin ELFA showed higher sensitivity and lower specificity (56.4%, CI, 42.4–69.5 and 95.4%, CI, 91.4–97.6, respectively) but they were not significant.

Most previous studies on GDH tests used ELISA kits [9]. Only a few, recent studies evaluated GDH ELFA, and GDH ELFA showed high sensitivity and NPV comparable to those of the Xpert *C*. *difficile*, suggesting that it is suitable for initial screening [22–24].

Our study demonstrated the high sensitivities and specificities of the Xpert *C*. *difficile* and both algorithms (GDH + Xpert and GDH + Toxin + Xpert). The performance of the Xpert *C*. *difficile* was comparable to those of recent reports (sensitivity, 94.4–100.0%; specificities, 93.0–98.8%) [18, 25, 26]. There was no significant difference in sensitivity, specificity, PPV, and NPV between the Xpert *C*. *difficile* and each algorithm. This finding demonstrates that we can choose either one in terms of performance (**Table 2**).

Regarding the costs, 68.6% of samples by GDH + Xpert and 78.6% by GDH + Toxin + Xpert had the cost per sample of $10 and $20, respectively, and the other samples submitted to the second step had higher cost per sample of $60 and $70, respectively (**Table 3**). The cost of Xpert *C*. *difficile* was about $50 per sample. Based on the distribution of our samples, the average cost per sample in the two algorithms was estimated to be $25.7 and $30.7, respectively; it was lower than that of the Xpert *C*. *difficile*. However, rapid detection of CDI by Xpert *C*. *difficile* may offset laboratory costs by reducing hospitalization and *C*. *difficile* transmission from the view of total medical costs [27].

In terms of assay time excluding preparation step, the Xpert *C*. *difficile* could report the results of each sample within an hour. The GDH + Xpert and GDH + Toxin + Xpert algorithms could report 68.6% and 78.6% of samples, within 50 min and 75 min, respectively; it was comparable to the Xpert *C*. *difficile*. For the other samples submitted to the second step, it took about twice of time. Comparing the two algorithms, GDH + Xpert had shorter assay time and sample cost, but GDH + Toxin + Xpert could report more samples at the first step.

The actual cost and turnaround time can vary across laboratories, according to the workflow, the number of requested tests, and many other factors. The Xpert *C*. *difficile* can be performed immediately on demand or several times per day according to the number of spaces for cartilages in instrument. For examples, simulated assay time per 30 samples (maximal daily number of requested tests in our hospital) would be extended in Xpert *C*. *difficile* when number of space for cartilages in GeneXpert system is limited, 4 cartilages in our system (**Table 3**). The system with more cartilages loading is needed for rapid reporting. The VIDAS system for GDH ELFA and toxin ELFA is also simple and easy to perform, offering routine batch or random access testing, whereas conventional ELISA system is difficult to perform as random access testing. Each laboratory can determine its own policy and can optimize the diagnostic workflow depending on its situations.

In this study, we evaluated the performances of assays and algorithms based on toxigenic culture as a reference standard, which is suggested by many experts and guidelines [1, 9]. Another reference standard is the cell cytotoxicity assay, and these two reference methods have different targets, toxigenic *C*. *difficile* isolates and direct toxin, respectively [4, 5]. A recent, multicenter study showed that toxin positivity by cytotoxicity assay more correlated with clinical outcomes, and toxigenic culture could detect the *C*. *difficile* excretes with diarrhea not due to CDI [4]. It also showed that GDH + NAAT and toxin EIA + NAAT best reproduced toxigenic culture and cell cytotoxin assay, respectively. Moreover, NAAT itself and confirmation by NAAT in the second step of algorithms could overdiagnose CDI [4, 9, 11]. Since toxin detection is still important and has a clinical impact, toxin assays with improved performances are expected. In addition to the detection itself, performances of assays and algorithms need to be evaluated along with their clinical significances in further studies.

In conclusion, both algorithms screening with GDH and/or toxin ELFA confirmed by the Xpert *C*. *difficile* showed comparable performances to that of the Xpert *C*. *difficile*. This data would be helpful to choose the diagnostic algorithms for the detection of CDI in clinical laboratories. Each laboratory can optimize its diagnostic workflow in accordance with its situation encompassing the costs, test volumes, and clinical needs.
